# Transition from Pediatric to Adult Care in Patients with Transfusion-Dependent Beta-Thalassemia in France: A National Study Concerning a Rare Disease

**DOI:** 10.3390/jcm15062203

**Published:** 2026-03-13

**Authors:** Sarah Szepetowski, Audrey Benoit, Julie Berbis, Catherine Badens, Consortium NaThalY, Estelle Jean, Benjamin de Sainte Marie, Imane Agouti, Isabelle Thuret

**Affiliations:** 1 Reference Center for Red Blood Cell Diseases, AP-HM, 13385 Marseille, France; 2 French National Thalassemia Registry (NaThalY), AP-HM, 13385 Marseille, France; 3 Pediatric Immuno-Hematology, AP-HM, 13385 Marseille, France; 4 Department of Public Health APHM Marseille, UR3279 CERESS, Aix Marseille University, 13385 Marseille, France; 5 Biochemistry Department, AP-HM, 13385 Marseille, France; 6 C2VN, INSERM, Aix Marseille University, AP-HM, 13385 Marseille, France; 7 Internal Medicine, AP-HM, 13385 Marseille, France

**Keywords:** beta-thalassemia, transition, pediatrics, young adults, iron overload, transfusion dependency

## Abstract

**Background/Objectives**: Transfusion-dependent β-thalassaemia (TDT) is a lifelong condition requiring coordinated multidisciplinary care. In France, where the disease is rare, transition from pediatric to adult care remains poorly structured, potentially compromising adherence and long-term outcomes. **Methods**: This national retrospective study evaluated current transition practices and their clinical impact among young adults with TDT. Patients aged 20–25 years in December 2022 were identified from the national NaThalY registry. Those diagnosed and managed in France before age 15 were included. Clinical data were collected for the two years preceding and following transition. Transition practices were assessed using a standardized questionnaire sent to pediatric centers. **Results**: Thirty-four patients were included (mean transition age: 19 years). The rate of response to the questionnaire was 90.5%, with feedback from 19 centers. Only one-third of centers offered joint pediatric–adult consultations, and one-quarter provided transition-focused education. No written transition protocols were reported. Mean pre-transfusion hemoglobin levels were significantly lower after the transition (8.5 vs. 8.0 g/dL; *p* = 0.01). Ferritin levels showed a non-significant increase, with no statistically significant changes observed in hepatic or cardiac iron concentrations. **Conclusions**: This study demonstrates marked heterogeneity and limited formalization of transition practices in France. Development of structured, standardized transition pathways is urgently needed to ensure continuity of care and optimal disease management in adults with TDT.

## 1. Introduction

Beta-thalassemias are chronic hereditary disorders caused by mutations in the beta-globin gene, leading to defective hemoglobin synthesis [1,2,3]. Beta-thalassemias are common in the Mediterranean region, the Middle East, India, China, and Southeast Asia but very rare in France, found mainly in populations that have migrated from the abovementioned regions. In France, the national registry (NaThalY) documents almost 800 patients living with β-thalassemia, with approximatively half of them being dependent on regular blood transfusions [4,5].

Each unit of transfused packed red blood cells contains approximately 200–250 mg of elemental iron. While physiological iron homeostasis is tightly regulated through controlled absorption, recycling, and storage mechanisms, largely governed by hepatic hormone hepcidin, the human body lacks a physiological mechanism for removal of the excess iron load. In transfusion-dependent patients, iron progressively accumulates, as erythropoietic needs are mainly met by macrophage-mediated recycling of senescent erythrocytes rather than dietary absorption. When the serum transferrin saturation is about 60–70%, exceeding the transferrin binding capacity, non-transferrin-bound iron (NTBI) appears in the circulation, promoting oxidative stress, cellular dysfunction, and tissue damage, thereby necessitating lifelong iron chelation therapy [6,7]. Therefore, iron overload remains the main cause of morbidity in β-thalassemia major, with complications predominantly affecting the cardiac, hepatic, and endocrine systems. When chelation is initiated early and maintained consistently, iron overload can be effectively prevented during childhood, allowing survival to exceed 50 years in many cases [4,8,9]. According to the French registry, approximately 250 adults with transfusion-dependent β-thalassemia are currently under medical care in France.

The transition from the pediatric team to the adult team is a key stage in the management of patients with chronic illnesses, as potential discontinuity of care can adversely affect health and lead to life-threatening complications. In 1993, the Society for Adolescent Medicine defined transition as an “active and multifaced process addressing the medical, psychosocial, educational, and vocational needs of adolescents as they move from a child-focused to an adult-focused healthcare system” [10]. The transition process, occurring during adolescence, and the physical and psychological changes it entails bring into play numerous social factors linked to the differences in functioning between the pediatric and adult worlds. Although there is no standardized framework for managing transition in chronic illness care, it typically occurs around the age of 18, with early preparation being strongly recommended [11,12]. Regarding β-thalassemia, historically a pediatric illness, few studies have focused on the transition [13,14,15,16]. It is well known that adherence to medical therapies begins to decline during adolescence [17]. In transfusion-dependent thalassemia, adherence to iron chelation medication is a key component of health outcomes due to the risks associated with iron overload. Adherence has been shown to have a variety of physiological effects, notably impacting cardiac outcomes, liver health and endocrine outcomes. Some factors, such as the use of oral iron chelators, have been identified as improving adherence, but the difficulty remains with respect to how to define it and measure it precisely [18].

In France, the transition from pediatric to adult care for patients with rare diseases, such as thalassemia, remains largely unstandardized. Despite national policy frameworks recognizing the need for structured transitions, published evidence shows that few centers implement dedicated transition coordinators or written protocols [19]. This lack of consistency poses serious risks: patients may be transferred without adequate preparation, leading to loss to follow-up, treatment non-adherence and sub-optimal outcomes. For chronic red-cell disorders like thalassemia, where lifelong care is essential, these gaps underscore an urgent need for national standards, dedicated transitional programs, and seamless coordination between pediatric and adult services.

The primary objective of this national retrospective study is to describe clinical practices surrounding the transition from pediatric to adult health care of transfusion-dependent β-thalassemia patients, documenting patient characteristics at the time of transition and outlining the general procedures implemented in healthcare centers to support the transition. Secondary objectives include studying changes in clinical management before and after transition (therapeutic adjustments, iron overload parameters, and organ damage) and evaluating the potential impacts of patient characteristics and transition procedures on the age at transition and evolution of iron overload levels.

## 2. Materials and Methods

Patients included in the study were identified from the French national registry, i.e., NaThalY, as of November 2024 and met the following criteria: a diagnosis of transfusion-dependent beta-thalassemia, residence in France, an age of 20–25 years as of 31 December 2022, and initiation of care in France before age 15. 

The French registry defines a patient as transfusion-dependent when he receives more than 8 transfusions per year, corresponding to regular transfusions. If regular transfusions begin before the age of 4, NaThalY classifies the patient as having Thalassemia Major; otherwise, the patient is classified as having Thalassemia Intermedia.

The 20–25 age group was chosen in an attempt to select the largest number of patients who had undergone transition, considering a minimum age of 18 at transition. Patients who underwent successful allogeneic bone marrow transplantation or gene therapy were excluded.

The study analyzed two years of follow-up in pediatric care preceding the transition and two years of adult care following the transition. Data analyzed in this study was directly extracted from the NaThalY registry, including the following variables: age at transition, sex, place of birth, vital status, ferritin levels, pre-transfusion hemoglobin concentrations, frequency of hepatic and cardiac MRI monitoring and associated iron concentrations, chelation and other treatments, organ damage, marital status, and educational or professional activity.

Practices surrounding transition were evaluated using a short, dedicated questionnaire sent to treating physicians. This questionnaire was developed by two medical experts in thalassemia, incorporating key elements of the transition according to the literature. Surveyed physicians were practicing at a center designated for clinical reference in thalassemia (reference center; RC) or at a non-reference center (non RC).

Statistical analyses were performed using SPSS software (version 17.0 for Windows). A significance threshold of 0.05 was used for all tests to determine statistical significance. Descriptive statistics were employed to summarize the data. Quantitative variables were characterized by their mean and standard deviation (for normally distributed data) or median and interquartile range (IQR; Q1–Q3) for skewed distributions. Qualitative variables were expressed as absolute frequencies and percentages. For categorical variables, associations were examined using the Chi-squared test. In cases of small sample sizes, Fisher’s exact test was used as an alternative. For continuous variables, group comparisons were conducted using a paired Student’s *t*-test for normally distributed data and a Wilcoxon signed-rank or Mann–Whitney U test for non-normally distributed data. For correlation analysis, Pearson’s correlation coefficient was calculated for parametric data, while Spearman’s rank correlation coefficient was used for non-parametric data.

## 3. Results

### 3.1. Patient Characteristics

**Figure 1:**
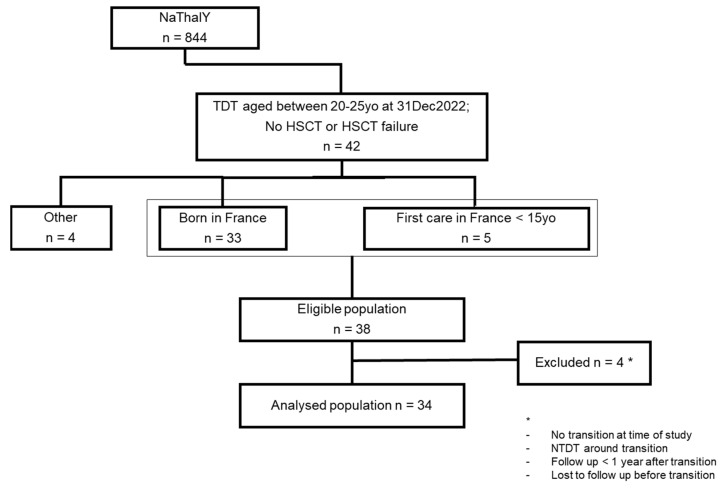
Flow chart of patients included in the study. (TDT: transfusion-dependent thalassemia; HSCT: hematopoietic stem cell transplant; yo: years old).

Overall, 34 patients were included in the study (flow chart, Figure 1), of whom 18 were female and 30 were born in France. The mean age at diagnosis was 1.7 years (min: 0; max: 6.3). Regarding genotypes, 61.8% were β0/β0, 17.6% were β0/β+ and 8.8% were β+/β+. One patient carried a heterozygous β+ mutation associated with a homozygous triplication of alpha genes. At the time of transition, 27 patients were managed in an RC. One patient underwent allogeneic stem cell transplantation, which ended in failure, which is why he was included. All patients were on a long-term transfusion program according to the transfusion dependency definition of the registry, and 14 patients (41%) had a splenectomy. Regarding chelation therapy, all patients received treatment at the three assessment points (Table 1).

Four patients were excluded (Figure 1), including a 20-year-old patient who had not transitioned at the time of enrolment and one patient with non-transfusion-dependent thalassemia (NTDT) who had received a transfusion regimen between 13 and 15 years old to achieve growth and pubertal development.

### 3.2. Clinical Practices Around Transition

Twenty-seven patients during the pre-transition period and 25 patients during the post-transition period were managed in a reference center for red blood cell disorders. To assess clinical practices around transition, a standardized questionnaire was sent to the 21 pediatric centers, with a response rate of 90.5%. Only 16% (n = 3) of centers offered a thalassemia-specific therapeutic education program or sessions. Concerning transition, 32% (n = 6) of centers proposed a shared consultation between the pediatric and adult teams, all implemented over the last 10 years, and 26% (n = 5) offered therapeutic education sessions focused on transition (Table 2).

Finally, six centers implemented at least one procedure targeting transition (joint consultation with pediatrician and the adult physician or therapeutic education program). Among the patients included in this study, 10 benefited from this management. No center reported the existence of written procedures specific to the transition. Electronic medical records were shared by the adult and pediatric teams in 16 centers (84.2%). Only six centers reported offering systematic psychological follow-up.

### 3.3. What Did We Observe Around the Transition?

The mean age at transition was 19 years old (min 17.5; max 21.3); two patients completed their transition before 18 years old, 18 patients between 18 and <19 years old, eight between 19 and <20 years old and six patients after 20 years old. Ten patients were followed in centers that offered therapeutic education sessions focused on transition or shared transition consultations (Table 1).

**Figure 2:**
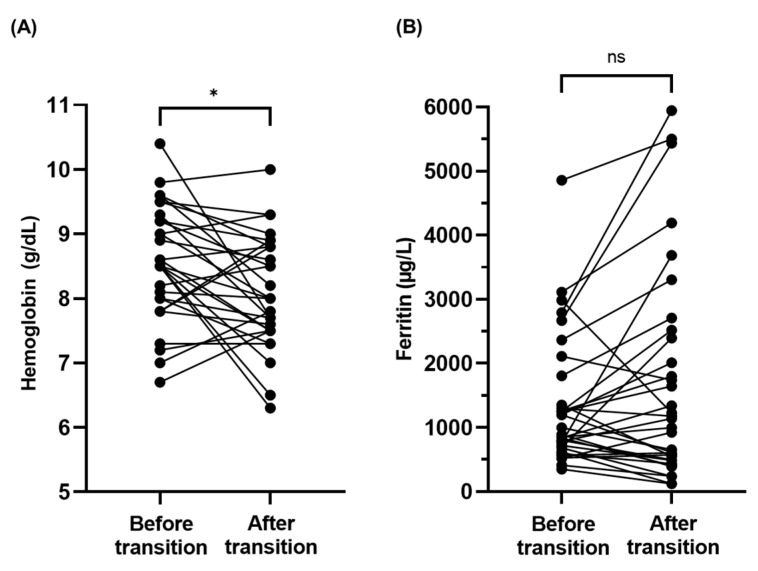
Evolution of paired hemoglobin (**A**) and ferritin (**B**) levels before and after transition. Each timepoint represents the two years preceding and following the date of transition. * significant *p*-value > 0.05; ns: non-significant *p*-value.

Pre-transfusion hemoglobin (Hb) did not vary in the two years leading to the transition but ultimately significantly decreased in the four years around transition (8.5 g/dL vs. 8.0 g/dL; *p* = 0.01) (Figure 2A).

Based on MRI examination, two-thirds (64.3%) of patients had no or low hepatic iron overload at transition. Ferritin increased in the four years around transition, from 1330.5 µg/L to 1684.5 µg/L (*p* = 0.153), without any significance, and there was no impact on liver and cardiac iron concentrations as evaluated by MRI (Figure 2B).

Regarding iron chelation, the most frequently used chelator was Deferasirox. Overall, ten patients received a combination of two iron chelators at some point in the four years surrounding transition, but only two patients remained under bitherapy for the entire study period, and the association of Deferasirox + Deferoxamine was the most common (Table 1).

Organ complications were found in seven (20.6%) patients in the two years leading to the transition, and six (17.6%) were still affected at the time of transition—all with endocrine disorders. Four patients had delayed puberty before transition that was no longer present at the time of transition. One patient with severe iron overload (ferritin level > 5000 µg/L and hepatic MRI = 17.5 mg/g) developed liver fibrosis (measured by transient elastography) within the two years following transition; the last assessment was seven years before transition (hepatic MRI = 19.7 mg/g and cardiac MRI = 12 ms). No death occurred during the study period (Table 1).

Regarding school and professional activity among patients with data available before the transition (28 patients), only one patient (3.6%) was neither in school nor employed. Two years after the transition, 15 patients were still students, five were employed and four patients were unemployed (Table 1).

### 3.4. Are There Any Factors Impacting Transition?

Regarding healthcare centers, there was no evidence of difference in Hb or ferritin levels between patients from RC and patients from non RC before transition. Fourteen patients (41%) had a follow-up in a different hospital or city after transition, without any impact on iron overload data.

We did not demonstrate significant changes in ferritin and Hb levels according to gender and the type of support provided during the transition.

There was no evidence of the effect of age at transition: among the six patients who had a transition after 20 years old, even though ferritins were higher in the post transition period (1415.17 µg/L 2 years before transition vs. 2011.67 µg/L 2 years after transition), the difference was not significant. There was also no significant difference in ferritin levels after transition between patients who did their transition before and after the age of 20.

We did not find any impact of gender, ferritin values or the level of transition support on age at transition.

## 4. Discussion

This national retrospective study highlights the heterogeneity and challenges of the transition process for patients with transfusion-dependent beta-thalassemia in France. Thalassemia remains a rare disease in France, with approximately 900 patients with symptomatic β-thalassemia recorded in the French National Registry (unpublished NaThalY data). Life expectancy has improved markedly in recent decades with better monitoring of iron overload and the advent of oral chelation therapy [20].

Transition from the pediatric to adult team is a critical stage in the management of patients with chronic disease. Young adults are faced with many challenges, including managing their illness while navigating other significant life changes, such as completing their education, starting employment, and forming new social relationships [21,22]. At the same time, parental involvement shifts. As the adolescent assumes greater responsibility for their care, they gain more autonomy, but this can be difficult to manage. Parents, who were once the primary managers of their child’s health care, may struggle to adjust to this new dynamic, creating tensions and challenges in finding a balance. This transformation can sometimes lead to conflicts or misunderstandings, especially when adolescents make decisions regarding their health without consulting or involving their parents, which is often compounded by their desire for independence [23,24]. Transfer to adult care is usually around the age of 18, which is similar to what we found in our study. However, some studies suggest the age of 18 may not be the best indicator of readiness and age should not be the sole criterion for leaving pediatric care [25]. Indeed, regarding the evaluation of the practices in healthcare centers, our findings show considerable variability. Only a minority of them offer transition-specific therapeutic education programs, and even fewer provide written protocols or structured pathways for thalassemia patients. Shared consultations between pediatric and adult teams are rare, and psychological support is not systematically provided, despite well-documented emotional and organizational vulnerabilities during this period [24,26,27]. We also found that there was little transmission of information at the paramedical level between pediatric and adult staff, which could impact the transition. Literature data show that successful transitions depend on several key factors, including patient readiness, parental support, and coordination between pediatric and adult healthcare teams [28]. Suris et al. identified six essential elements for effective transition programs, stressing the importance of collaboration between healthcare teams and uninterrupted patient follow-up [27]. In 2016, Oswald et al. identified female gender and maternal education as having a significant impact on the success of a transition [29]; in our study, we found no impact of gender on Hb and ferritin levels around transition and no impact of gender on age at transition. Barriers to successful transitions include issues related to patients’ lack of self-management skills, parental reluctance to relinquish control, and the differences between pediatric and adult care systems. Studies have shown that proper training and support prior to transition can help adolescents take an active role in managing their condition [11,13,30].

Few studies have focused on transition in thalassemic patients compared to sickle cell disease, for which there is more data available due to the much higher incidence of this disease [31,32,33,34,35,36]. A 2023 study on sickle cell disease patients aged 18 to 24 found that those who missed their first adult care appointment or had no pediatric follow-up for two years before age 18 shared characteristics such as unaltered treatment plans during their last pediatric visit, care in non-specialized centers, and being lost to follow-up before age 15 [37]. Studies such as that by Wojciechowski et al. have shown that participation in transition programs improves attendance at sickle cell disease adult care follow-ups [38]. These programs, which provide education and coordinated care between pediatric and adult teams, help ensure continuity of treatment and patient engagement. Research by Howell [34] and Rea [33] highlights that such programs lead to better health outcomes by fostering self-management skills and adherence to treatment, emphasizing the importance of early preparation and continuous care to support a successful transition.

In transfusion-dependent β-thalassemia, optimization of transfusion intensity and iron chelation remains a cornerstone of long-term outcomes and quality of life [39,40]. Although pediatric and adult teams had access to shared electronic medical records for most patients in our cohort, facilitating information transfer, we observed a significant decline in pre-transfusion hemoglobin levels after transition, associated with an increase in ferritin concentrations, without a measurable impact on cardiac or hepatic iron overload assessed by MRI. Several potentially overlapping mechanisms may explain this decline in hemoglobin levels. First, transfusion targets in adult care are often lower than those applied in pediatric practice, reflecting differences in clinical culture, perceived tolerance to chronic anemia, and concerns regarding transfusion-related complications [39,41]. Second, reduced adherence to transfusion schedules and/or chelation therapy after transition may play a role, as patients assume greater autonomy and face competing educational, professional, and psychosocial demands [42]. For instance, the need for intensification of iron chelation appeared from the moment of transition and afterwards, with bitherapy being prescribed in 20% of the study population two years after transition, when it was in only prescribed in 8% of the population two years prior, consistent with the increase in ferritin levels by that time. Finally, limited familiarity of some adult providers with thalassemia-specific guidelines, particularly regarding transfusion thresholds and iron monitoring, has been reported and may influence clinical decision-making [14]. Our findings are consistent with those of Stacy et al., who identified substantial variability and knowledge gaps in adult thalassemia care in the United States, particularly with respect to transfusion targets and iron surveillance. Importantly, only a limited number of centers in our study implemented structured transition interventions, such as joint pediatric–adult consultations or formal educational programs, limiting comparative analyses. Nevertheless, the sustained decrease in pre-transfusion hemoglobin observed at long-term follow-up—up to seven years after transition for some patients—together with a significant increase in hepatic iron burden compared with the pre-transition period, suggests that these changes are not transient. Rather, they may reflect durable shifts in management practices and/or patient engagement after transfer to adult care. Future prospective studies should aim to disentangle these mechanisms by systematically comparing transfusion targets, adherence metrics, organizational factors, and provider expertise across pediatric and adult settings. Incorporation of patient-reported outcomes, health literacy assessments, and evaluation of structured transition programs will be essential to define evidence-based transition models capable of maintaining optimal hemoglobin levels and iron control into adulthood [42].

## 5. Conclusions

In conclusion, our study highlighted differences in care and practice around the transition of patients with transfusion-dependent thalassemia on a national scale. The lack of specific procedures and therapeutic education programs to support the transition is an obstacle to the success of this key stage in the patient care pathway. On the other hand, disparities in knowledge of this historically pediatric condition could impact the transition. It therefore seems necessary to harmonize practices—in particular, by writing a national transition procedure to provide optimal lifelong management for these patients. Thereafter, prospective studies could assess the impact of transition on medication compliance, arising long-term complications and quality of life.

## Figures and Tables

**Table 1:** Characteristics of study population around transition.

Study Population, n	34			
**Sex, n (%)**				
Male	16 (47.1)			
Female	18 (52.9)			
Genotypes				
β0/β0	21 (61.8)			
β0/β+	6 (17.6)			
β+/β+	3 (8.8)			
Other	1 (2.9)			
Unknown	3 (8.8)			
	**2 years before** **Transition**	**At transition**	**2 years after** **transition**	*p*-*value*
**Age, years**				
Mean (SD)	17.1 (0.9)	19.1 (0.9)	21.1 (0.9)	
**Treatment, n (%)**				
Transfusion program	34 (100)	34 (100)	34 (100)	
Splenectomy	13 (38.2)	13 (38.2)	14 (41.2)	
HSCT *		1 (2.9)		
Chelation therapy	34 (100)	34 (100)	34 (100)	
Monotherapy	31 (91.2)	27 (79.4)	26 (76.5)	
Bitherapy	3 (8.8)	7 (20.6)	8 (23.5)	
**Pre-transfusion Hb (g/dL)**	n = 31	n = 31	n = 33	
Mean (SD)	8.56 (0.88)	8.57 (1.03)	8.06 (0.82)	**0.010**
*p*-*value* (compared to transition)	0.490		**0.027**	
**Iron overload at transition**				
Ferritin, µg/L	n = 34	n = 31	n = 33	
Mean (SD)	1301 (1001)	1433 (1345)	1685 (1646)	0.153
*p-value* (compared to transition)	0.085		0.217	
Hepatic MRI, n (%)	n = 28 (82.3)		n = 23 (67.6)	
≤3 mg/g	10 (35.7)		7 (30.4)	
>3 and ≤7 mg/g	8 (28.6)		5 (21.7)	
>7 and ≤15 mg/g	7 (25.0)		6 (26.1)	
>15 mg/g	3 (10.7)		5 (21.7)	
Mean, mg/g (SD)	8.3 (9.5)		9.2 (9.3)	0.542
Cardiac MRI, n (%)	22 (64.7)		20 (58.8)	
No iron overload (T2* > 20 ms)	20 (90.9)		17 (85)	
Iron overload (T2* < 20 ms)	2 (9.1)		3 (15)	
**Organ damage at transition**				
Patients with complications, n (%)	7 (20.6)	6 (17.6)	6 (17.6)	
Liver fibrosis	0	*0*	*1*	
Diabetes	1	1	1	
Hypogonadism	2	2	2	
Hypoparathyroidism	2	2	1	
Osteoporosis	4	2	2	
**Social data at transition, n (%)**				
Studying/working	n = 27 26 (96.3)	n = 28 27 (96.4)	n = 24 20 (83.3)	
Parenthood (n = 30)	1 (3.3)	1 (3.3)	1 (3.3)	

* graft failure. HSCT: Hematopoietic Stem Cell Transplant; Hb: Haemoglobin; MRI: Magnetic Resonance Imaging. Statistically significant *p*-value > 0.05.

**Table 2:** Assessment of practices around transition in France.

	Yes, n (%)	No, n (%)
Therapeutic education sessions for transition	5/19 (26)	14/19 (74)
Therapeutic education sessions for thalassemia patients	3/19 (16)	16/19 (84)
Written procedures on transition for thalassemia patients	0	19 (100)
Systematic psychological follow-up during transition	6/19 (32)	13/19 (68)
Pediatric and adult departments located in the same hospital	14/19 (74)	5/19 (26)
Shared electronic medical records	16/19 (84)	3/19 (16)
Shared consultation between pediatric and adult physicians	6/19 (32)	13/19 (68)

A standardized yes/no questionnaire was sent to 21 different clinicians following the patients included in the study, of whom 19 responded and answered all questions.

## Data Availability

Restrictions apply to the availability of these data. Data were obtained from NaThalY (the French national thalassemia registry) and are available from the authors with the permission of NaThalY.

## Abbreviations

The following abbreviations are used in this manuscript:

TDT	Transfusion-dependent thalassemia
NTDT	Non-transfusion-dependent thalassemia
RC	Reference center
HSCT	Hematopoietic stem cell transplant
YO	Years old
Hb	Hemoglobin
MRI	Magnetic resonance imaging

## Appendix A

The following investigators contributed to NaThalY, composing the NaThalY Network:

Catherine Mathey, CH du Pays d’Aix, Aix en Provence; Louisa Bey, CH Notre-Dame de la Miséricorde, Ajaccio; Marie-Josée Lucchini, CH Notre-Dame de la Miséricorde, Ajaccio; Valérie Li Thio Te, CHU Amiens Picardie, Amiens; Reda Garidi, CHU Amiens Picardie, Amiens; Loïc Garcon, CHU Amiens Picardie, Amiens; Corentin Orvain, CHU d’Angers, Angers; Jean-François Brasme, CHU d’Angers, Angers; Claire Reynes, CH Annecy Genevois, Annecy; Marie Belloy, CHI Robert Ballanger, Aulnay-Sous-Bois; Julie Durin, CHI Robert Ballanger, Aulnay-Sous-Bois; Oula Alhindy, CH Henri Mondor, Aurillac; Lara Sabbagh, CH Henri Mondor, Aurillac; Borhane Slama, CH Henri Duffaut, Avignon; Anthony Sanchez, CH de Bastia, Bastia; Alice Moulin, CH de Bastia, Bastia; Danielle Belgodere, CH de Bastia, Bastia; Lise Benjemia, CHRU Besançon, Besancon; Pauline Simon, CHRU Besançon, Besancon; Eric Oziol, CH de Béziers, Beziers; Nathalie Aladjidi, Groupe hospitalier Pellegrin, Bordeaux; Yoann Huguenin, Groupe hospitalier Pellegrin, Bordeaux; Charlotte Jubert, Groupe hospitalier Pellegrin, Bordeaux; Edouard Forcade, Groupe hospitalier Pellegrin, Bordeaux; Pierre Duffau, Groupe Hospitalier Saint André, Bordeaux; Abdallah Maakaroun, CH Jacques-Coeur, Bourges; Florence Lachenal, Hôpital Pierre Oudot, Bourgoin-Jallieu; Charline Pegon, Hôpital d’Estaing, Clermont-Ferrand; Marie-Helene Odievre, CH Louis Mourier, Colombes; Charlotte Petitdidier, CHSF, Corbeil-Essonnes; Sai Atmani, CH Laennec, Creil; Florence Raqbi, CH Laennec, Creil; Corinne Pondarre, CHI Créteil, Créteil; Jenny Youn, CHI Créteil, Créteil; Annie Kamdem, CHI Créteil, Créteil; Dora Bachir, CHU Henri Mondor, Créteil; Pablo Bartolucci, CHU Henri Mondor, Créteil; Gonzalo De Luna, CHU Henri Mondor, Créteil; Anoosha Habibi, CHU Henri Mondor, Créteil; Elena Fois, CHU Henri Mondor, Créteil; Antoine Leblanc, CHSF, Evry; Pierre-Yves Dides, CH de Grasse, Grasse; Caroline Makowski, CHU Grenoble, Grenoble; Corinne Armari, CHU Grenoble, Grenoble; Narcisse Elenga, CH Ouest guyanais, Guyane; Christelle Chantalat-Auger, Hôpital Bicêtre, Le Kremlin-Bicêtre; Françoise Driss, Hôpital Bicêtre, Le Kremlin-Bicêtre; Ferielle Zenchri, Hôpital Bicêtre, Le Kremlin-Bicêtre; Corinne Guitton, Hôpital Bicêtre, Le Kremlin-Bicêtre; Mathilde Jehanne, CHU de La Réunion, La Réunion; Cecile Stoven, CHU de La Réunion, La Réunion; Axel Chaminade, CHU de La Réunion, La Réunion; Stephanie Gourdon, CHU de La Réunion, La Réunion; Yves Reguerre, CHU de La Réunion, La Réunion; Edouard Randriamalala, CHU de La Réunion, La Réunion; Véronique Paumier-Bonjour, CH Jacques Monod, Le Havre; Anne Besancon-Bergelin, CH Le Mans, Le Mans; Anne Lambilliotte, CHRU Jeanne de Flandre, Lille; Benedicte Bruno, CHRU Jeanne de Flandre, Lille; Fanny Gonzales, CHRU Jeanne de Flandre, Lille; Melissa Barbati, CHRU Jeanne de Flandre, Lille; Benjamin Carpentier, Hopital Saint Vincent de Paul, Lille; Christophe Piguet, CHU Dupuytren, Limoges; Mohamed Touati, CHU Dupuytren, Limoges; Arnaud Hot, CHU Edouard Herriot, Lyon; Fiorenza Barraco, CHU Edouard Herriot, Lyon; Giovanna Cannas, CHU Edouard Herriot, Lyon; Emilie Virot, CHU Edouard Herriot, Lyon; Romain Fort, CHU Edouard Herriot, Lyon; Grégoire Ducoux, CHU Edouard Herriot, Lyon; Hélène Desmurs Clavel, CHU Edouard Herriot, Lyon; Kamila Kebaili, Institut d’hématologie et d’oncologie pédiatrique, Lyon; Nathalie Garnier, Institut d’hématologie et d’oncologie pédiatrique, Lyon; Cécile Renard, Institut d’hématologie et d’oncologie pédiatrique, Lyon; Yves Bertrand, Institut d’hématologie et d’oncologie pédiatrique, Lyon; Anne-Laure Peugnet, Institut d’hématologie et d’oncologie pédiatrique, Lyon; Carine Halfon-Domenech, Institut d’hématologie et d’oncologie pédiatrique, Lyon; Judith Diakiesse, CH François Quesnay, Mantes La Jolie; Kokou-Placide Agbo-Kpati, Grand hôpital de l’Est Francilien, Marne la Vallée; Nathalie Ravet, Grand hôpital de l’Est Francilien, Marne la Vallée; Isabelle Thuret, Hôpital de la Timone, Marseille; Estelle Jean, Hôpital de la Timone, Marseille; Benjamin De Sainte Marie, Hôpital de la Timone, Marseille; Benoit Meunier, Hôpital de la Timone, Marseille; Benoit Faucher, Hôpital de la Timone, Marseille; Matias Muszlak, CH Mayotte, Mayotte; Abdourahim Chamouine, CH Mayotte, Mayotte; Alain Garou, CH Mayotte, Mayotte; Caroline Masserot-Lureau, Grand hôpital de l’Est Francilien, Meaux; Jean-Marc Schneider, CH Bel-Air Metz Thionville, Metz-Thionville; Veronique Dorvaux, CHR Metz Thionville, Metz-Thionville; Philippe Carassou, CHR Metz Thionville, Metz-Thionville; Honorat Andriamihaja, Centre Hospitalier de l’Agglomération Montargoise, Montargis; Muriel Lalande, Hôpital Arnaud de Villeneuve, Montpellier; Perrine Mahe, Hôpital Arnaud de Villeneuve, Montpellier; Anne Sirvent, Hôpital Arnaud de Villeneuve, Montpellier; Patricia Aguilar Martinez, Hôpital Arnaud de Villeneuve, Montpellier; Ivan Bertchansky, Hôpital Arnaud de Villeneuve, Montpellier; Jean-Claude Eisenmann, CH Mulhouse Emile Muller, Mulhouse; Agathe Masseau, CHU de Nantes, Nantes; Marie-Laure Couec, CHU de Nantes, Nantes; Fanny Rialland, CHU de Nantes, Nantes; Pierre-Simon Rohrlich, CHU Archet, Nice; Sandrine Baron-Joly, CHU de Nîmes, Nimes; Mohamed Conde, CHR Orléans, Orleans; Antoine Dossier, Bichat, Paris; Valentine Brousse, Hôpital Robert Debré, Paris; Karima Yakouben, Hôpital Robert Debré, Paris; Malika Benkerrou, Hôpital Robert Debré, Paris; Ghislaine Ithier, Hôpital Robert Debré, Paris; Florence Missud, Hôpital Robert Debré, Paris; Jean-Hugues Dalle, Hôpital Robert Debré, Paris; Bérengène Koehl, Hôpital Robert Debré, Paris; Laurent Holvoet, Hôpital Robert Debré, Paris; Jean-Antoine Ribeil, Hôpital Necker, Paris; Laure Joseph, Hôpital Necker, Paris; Marina Cavazzana, Hôpital Necker, Paris; Marianne De Montalembert, Hôpital Necker, Paris; Benedicte Neven, Hôpital Necker, Paris; Despina Moshous, Hôpital Necker, Paris; Mélissa Taylor, Hôpital Necker, Paris; Slimane Allali, Hôpital Necker, Paris; Bruno Varet, Hôpital Necker, Paris; Gerard Socie, CHU Saint Louis, Paris; Alienor Xhaard, CHU Saint Louis, Paris; Régis Peffault De La Tour, CHU Saint Louis, Paris; Thierry Leblanc, CHU Saint Louis, Paris; Nicolas Boissel, CHU Saint Louis, Paris; Marie Robin, CHU Saint Louis, Paris; Nathalie Dhedin, CHU Saint Louis, Paris; Robert Girot, CHU Tenon, Paris; Sarah Mattioni, CHU Tenon, Paris; François Lionnet, CHU Tenon, Paris; Jean Donadieu, CHU Trousseau, Paris; Guy Leverger, CHU Trousseau, Paris; Sylvie Fasola, CHU Trousseau, Paris; Jean-Benoit Arlet, Hôpital Européen George Pompidou, Paris; Corina Manoli, Hôpital Européen George Pompidou, Paris; Maryse Etienne-Julan, CHU de la Guadeloupe, Pointe à Pitre; Emmanuelle Bernit, CHU de la Guadeloupe, Pointe à Pitre; Justine Gellen-Dautremer, CHU la Milétrie, Poitiers; Stéphanie Eyssette, Hôpital NOVO, Pontoise; Gilles Blondin, Centre Hospitalier de Cornouaille, Quimper; Claire Pluchart, American Memorial Hospital, Reims; Stanislas Nimubona, Hôpital Pontchaillou, Rennes; Jacinthe Bonneau, Hôpital Sud, Rennes; Sophie Bayart, Hôpital Sud, Rennes; Sophie Pertuisel, Hôpital Sud, Rennes; Benedicte Collet, Hôpital Victor Provo, Roubaix; Laurence Detourmignies, Hôpital Victor Provo, Roubaix; Julie Machin, Hôpital Victor Provo, Roubaix; Mathieu Wemeau, Hôpital Victor Provo, Roubaix; Laurence De Tourmigni, Hôpital Victor Provo, Roubaix; Jean-Pierre Vannier, CHU Charles Nicolle, Rouen; Bruno Filhon, CHU Charles Nicolle, Rouen; Cecile Dumesnil, CHU Charles Nicolle, Rouen; Agnes Lahary, CHU Charles Nicolle, Rouen; Pascale Schneider, CHU Charles Nicolle, Rouen; Stéphanie Ngo, CHU Charles Nicolle, Rouen; Aurore Malric, Hôpital Delafontaine, Saint Denis; Maud Schoeny, CH Saint Dizier, Saint Dizier; Claire Berger, CHU Nord Saint Etienne, Saint-Etienne; Isabelle Guichard, CHU Nord Saint Etienne, Saint-Etienne; Aurélie Desbree, CHU Nord Saint Etienne, Saint-Etienne; Jean-Louis Stephan, CHU Nord Saint Etienne, Saint-Etienne; Audrey David, Hôpital de Hautepierre, Strasbourg; Catherine Paillard, Hôpital de Hautepierre, Strasbourg; Alexandra Salmon, Hôpital de Hautepierre, Strasbourg; Shanti Ame, Nouvel Hôpital Civil, Strasbourg; Philippe Gestas, Clinique Cordella, Tahiti; Marianne Besnard, Clinique Paofai, Tahiti; Marie-Pierre Castex, CHU des Enfants, Toulouse; Geneviève Plat-Wilson, CHU des Enfants, Toulouse; Pierre Cougoul, CHU des Enfants, Toulouse; Marlène Pasquet, CHU des Enfants, Toulouse; Jill Serre, CHU Tours, Tours; Marion Yvert, CHU Tours, Tours; Jean-Baptiste Valentin, CHU Tours, Tours; Francis Duchene, Hôpital Nord Franche-Comté, Trévenans; Suzanne Mathieu, CHU de Nancy—Hopital Brabois, Vandoeuvre Les Nancy; Marie Drouilly, CHU de Nancy—Hopital Brabois, Vandoeuvre Les Nancy; Marie Detrait, CHU de Nancy—Hopital Brabois, Vandoeuvre Les Nancy; Cécile Pochon, CHU de Nancy—Hopital Brabois, Vandoeuvre Les Nancy; Aurélie Phulpin, CHU de Nancy—Hopital Brabois, Vandoeuvre Les Nancy; Sylvie Nathanson, Centre Hospitalier de Versailles, Versailles.
